# Efficacy of recombinant human bone morphogenetic protein-2 for bone regeneration in alveolar defects: A protocol for systematic review and meta-analysis

**DOI:** 10.1097/MD.0000000000031958

**Published:** 2022-12-09

**Authors:** Xudong Ma, Xueqi Ju, Juehui Shen, Lala Zheng

**Affiliations:** a Department of Dentistry, Huzhou University, Huzhou, China; b Famous Doctor Hall, Hangzhou Dental Hospital, Hangzhou, China; c Department of Periodontology, Hangzhou Dental Hospital, Hangzhou, China; d General Department, Hangzhou Dental Hospital Group Nanxun Dental Clinic, Huzhou, China.

**Keywords:** alveolar defects, bone regeneration, meta-analysis, protocol, Recombinant human bone morphogenetic protein 2, systematic review

## Abstract

There is still a lack of credible evidence in the literature regarding the effectiveness of recombinant human bone morphogenetic protein 2 (rhBMP-2) in bone regeneration of alveolar bone defects. Therefore, the purpose of this study is to evaluate the efficacy and safety of rhBMP-2 in autogenous bone graft in bone regeneration of alveolar bone defects and to provide basis for clinical practice.

**Methods:**

As of March 2023, two independent investigators will identify target literature by searching seven major databases (EMBASE, Google Scholar, PubMed, Cochrane Library, Wan Fang, CNKI, and Web of Science). All clinical cohort studies evaluating the efficacy of rhBMP-2 will be included. The outcomes of the study include changes in the depth of the dental pocket at the involved site, increased clinical attachment levels, patient satisfaction, and adverse events. The Cochrane risk of bias tool will be independently used to evaluate the risk of bias of included randomized cohort studies by two reviewers. A modified version of the Downs and Black tool is adopted to evaluate the quality of nonrandomized cohort studies.

**Results:**

We hypothesized that rhBMP-2 plays an active role in bone regeneration in alveolar bone defects.

**Conclusions:**

It is worthy to critically review the evidence of the assessment of rhBMP-2 to inform clinical practice.

## 1. Introduction

Alveolar bone loss is a frequently encountered problem faced in orthodontic treatment, which is mainly caused by removal of adjacent teeth, surgical orthodontic treatment and severe periodontitis. Autologous tissue has been the gold standard for alveolar bone defect repair due to its bone-conducting and bone-inducing properties. Currently, the most widely accepted donor site is the ilium, which has a graft bone survival rate of 41% to 73%.^[1,2]^ However, donor site defects, potential infection, surgical trauma, and even morbidity are limiting factors in the harvesting of autogenous bone grafts. As the need for cancellous bone use on both sides of the ilium increases, the risk of donor site complications increases accordingly.^[3–5]^

Bone morphogenetic proteins are a group of bioactive proteins with osteoinductive properties that are part of the transforming growth factor superfamily. The literature has shown that the ß superfamily of transforming growth factors, including recombinant human bone morphogenetic protein 2 (rhBMP-2), plays an important role in mediating bone formation and repair by recruiting and differentiating osteoblasts and osteoclasts, which initiate the initial process of bone grafting.^[6–8]^ The use of rhBMP-2 in the reconstruction of large mandibular defects secondary to extensive tumors in the animal mandible and in clinical trials has shown promising results.^[9,10]^ However, recent data have shown that when 1.5 to 4.2 mg of rhBMP-2 is used to repair alveolar cleft, significant edema and gingival spasm occur, which may affect upper lip anatomy and alter soft tissue relationships.^[11]^

There is still a lack of credible evidence in the literature regarding the effectiveness of rhBMP-2 in bone regeneration of alveolar bone defects. Therefore, the purpose of this study is to evaluate the efficacy and safety of rhBMP-2 in autogenous bone graft in bone regeneration of alveolar bone defects and to provide basis for clinical practice. We hypothesized that rhBMP-2 plays an active role in bone regeneration in alveolar bone defects.

## 2. Materials and Methods

The protocol of systematic literature review and meta-analysis was structured to adhere to the PRISMA (Preferred Reporting Items for Systematic Reviews and Meta-Analyses) guidelines, which include requirements deemed essential for the transparent reporting of results.^[12]^ The systematic review protocol was registered on PROSPERO (CRD42022368802). Ethical approval was not necessary because the present meta-analysis was performed on the basis of previous published studies.

### 2.1. Selection of studies

**Figure 1.**
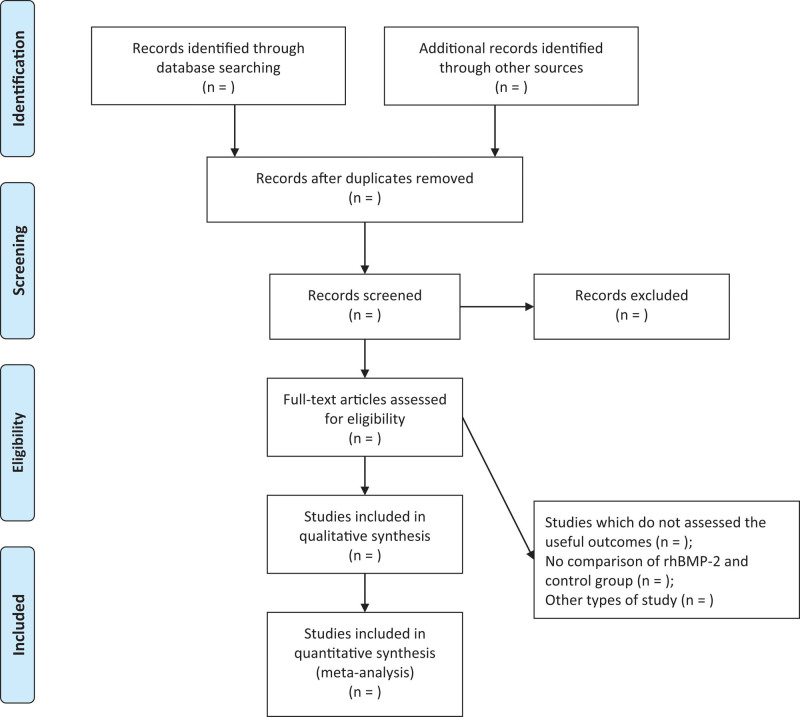
PRISMA flow diagram.

As of March 2023, two independent investigators will identify target literature by searching seven major databases (EMBASE, Google Scholar, PubMed, Cochrane Library, Wan Fang, CNKI, and Web of Science). The following search terms were used to search electronic databases to ensure that all possible relevant articles were included: (alveolar defect or alveolar cleft or alveolar reconstruction or alveolar augmentation) and (recombinant human bone morphogenetic protein-2 or rhBMP-2). In addition, references and citations of eligible literature will also be manually screened to further reduce the possibility of omissions. There are no language restrictions for inclusion (Fig. 1).

### 2.2. Inclusion and exclusion criteria

Study included in this systematic review and meta-analysis has to meet all of the following inclusion criteria:

Population: adult patients with alveolar defects;

Intervention: group with rhBMP-2;

Comparator: group without rhBMP-2;

Outcomes: the outcomes of the study include changes in the depth of the dental pocket at the involved site, increased clinical attachment levels, patient satisfaction, and adverse events.

Study design: clinical cohort studies

The exclusion criteria are as follows: studies which do not assessed the above outcomes; no direct comparison of rhBMP-2 and controls; studies with the following types: case reports, comments or letters, biochemical trials, protocols, conference abstracts, and reviews.

### 2.3. Study selection and data extraction

After removing duplicate search terms, two reviewers will screen titles and abstracts for studies that meet the eligibility criteria above. Subsequently, the remaining records will be submitted for full-text evaluation. During this step, the reference list of each manuscript will be reviewed for relevant citations. All electronic database screening, data extraction, and quality assessment will be performed in duplicate by two independent researchers. Differences that cannot be resolved after mutual discussions and revisions will be considered by the third researcher.

A customized data extraction table will be developed for the outcome. The lead author useds only published data for data extraction. The following information is mandatory: authors, year of publication, number of patients assigned to the rhBMP-2 treatment and control groups, patient characteristics such as age, gender, Body Mass Index and ethnicity, treatment details such as duration, treatment status and drug dose, follow-up period and outcome measures. In studies where data is missing or unavailable for meta-analysis or where data is presented only graphically, an attempt will be made to contact the corresponding authors by e-mail. Otherwise, we collect data according to the guidelines in the Cochrane Handbook for Systematic Reviews of Interventions. If necessary, the extraction of incomplete data will be abandoned. When disagreements in the collection of data arise, they will be resolved through discussion.

### 2.4. Risk of bias assessment

The Cochrane risk of bias tool will be independently used to evaluate the risk of bias of included randomized cohort studies by two reviewers.^[13]^ The quality will be assessed by using following seven items: random sequence generation, allocation concealment, blinding of participants and personnel, blinding of outcome assessment, incomplete outcome data, selective reporting, and other bias. A modified version of the Downs and Black tool is adopted to evaluate the quality of nonrandomized cohort studies.^[14]^ The modified version consists of 27 items with a total possible score of 29. A score of ≥75% indicates high quality, 60% to 74% indicates moderate quality and ≤60% low quality. Two investigators independently evaluate included studies on the 27 criteria, with any discrepancies resolved by a third independent reviewer.

### 2.5. Data synthesis

The present study will be performed by Review Manager Software (RevMan Version 5.3, The Cochrane Collaboration, Copenhagen, Denmark). We use the Mantel–Haenzel method to calculate the pooled odds ratio. Odds ratio with a 95% confidence interval is assessed for dichotomous outcomes. *P* < .05 is set as the significance level. The heterogeneity is assessed by using the *Q* test and *I*^2^ statistic. When *I*^2^ ≥ 40%, it is considered to represent significant heterogeneity. All outcomes are pooled on random-effect model. The *Z* test is used to assess the overall effect. The publication bias will be assessed by using funnel plots diagram. The funnel plot asymmetry will be evaluated by an Egger’s linear regression test to reveal any possible publication bias. Sensitivity analyses will be undertaken to determine the potential source of heterogeneity when significant.

## 3. Discussion

In general, clinical trials in humans and animals have shown a positive effect of rhBMP-2 on bone regeneration.^[6]^ It has been shown that the higher the dose of rhBMP-2, the more positive the effect on bone enhancement.^[15]^ The following limitations can be foreseen in this study: the quality of the included literature may be mixed and the sample size is relatively small; the dose of rhBMP-2 is diverse across studies and the optimal effective dose depends on the type and location of the bone and the carrier binding properties; the follow-up period may be focused on the short term and data for medium- and long-term follow-up are limited. Even so, this paper will be the first meta-analysis to comprehensively evaluate the efficacy and safety of rhBMP-2 for bone regeneration in the repair of alveolar bone defects with autologous bone grafts. We hypothesized that rhBMP-2 plays an active role in bone regeneration in alveolar bone defects.

## Author contributions

**Conceptualization:** Juehui Shen.

**Data curation:** Xudong Ma, XueqiJu.

**Investigation:** Xudong Ma, Xueqi Ju.

**Methodology:** Juehui Shen.

**Supervision:** Lala Zheng.

**Funding acquisition:** Jianyuan Wang.

**Writing – original draft:** Xudong Ma.

**Writing – review & editing:** Lala Zheng.
